# Heterogeneity of monogenic epilepsy in loci, phenotypes, and treatment approaches

**DOI:** 10.1007/s10072-026-08936-2

**Published:** 2026-03-31

**Authors:** Ali K. Saad, Nadia Akawi

**Affiliations:** Department of Genetics and Genomics, College of Medicine and Health Sciences, United Arab Emirates University, P. O. Box 1555, Al Ain, United Arab Emirates

**Keywords:** Seizures, Epilepsy, Neurodevelopment, Gene, Variants, Antiseizure medications

## Abstract

**Background:**

Monogenic epilepsies are a group of rare disorders that manifest in early childhood and are usually associated with neurodevelopmental abnormalities and poor drug response. The field of genetic epilepsy is continuously evolving in alignment with the vast advancements in genetic testing in the last decade.

**Methods:**

Recent studies and databases were reviewed to explore the latest updates in monogenic epilepsies.

**Findings:**

This literature review reveals the remarkable progress in understanding monogenic epilepsy classification, genetic aetiologies, and treatment over the past decade. The International League Against Epilepsy classification system continues to evolve, providing increasingly precise diagnostic frameworks that incorporate genetic and etiologic information. The genetic landscape of epilepsy has expanded dramatically, with around 2000 genes now associated with seizures and/ or epilepsy pertaining to complex and overlapping neuronal networks. Besides the locus heterogeneity, monogenic epilepsy phenotypes exhibit phenotypic variabilities due to differences in pathogenic causative variants as well as unique genetic backgrounds in affected individuals. Identification of the underlying molecular aetiology permits personalized management approaches which are currently limited approved agents. Genes encoding antiepileptic drug targets, metabolic enzymes and transporters are additionally implicated in the precision medicine.

**Conclusion:**

Current knowledge in Locus heterogeneity should be addressed in epilepsy phenotypes to avoid extensive diagnostic delays particularly in cases that can be rescued with available management options.

**Supplementary Information:**

The online version contains supplementary material available at https://doi.org/10.1007/s10072-026-08936-2.

## Epilepsy definition, epidemiology and classification

Epilepsy is the most common chronic neurological disorder, estimated to affect around 1% of the population in the globe [1–5]. Mortality rates among epilepsy patients are higher than in the general population, with sudden unexpected death occurring in a yearly incidence rate of 1.2 per 1000 individuals [5]. Epilepsy is a unified term describing heterogeneous phenotypes characterized by recurrent epileptic seizures that result from abnormal electrical activity in the brain. Epilepsy diagnosis is confirmed in cases having at least two unprovoked seizures separated by a 24-hour interval or one unprovoked seizure with a high probability of having further seizure episodes [6]. It can occur at any age, but predominantly affects children who are at higher risk of having abnormalities in brain development caused by or associated with epilepsy [7]. Clinical presentations of epilepsy phenotypes vary in electroclinical features, including seizure types, age of seizure onset, electroencephalographic findings, brain morphology, and/or underlying aetiology [8–10].

Syndromic and non-syndromic phenotypes contribute to the clinical landscape of epilepsy. Epilepsy phenotypes with characteristic electroclinical features are classified into syndromes to facilitate diagnosis (Table 1) [11]. Age of onset is one of the features that aid in the differential diagnosis of epilepsies. For instance, EIDEE manifest very early before 3 months of age unlike IESS that have infantile onset [8]. EIDEE display different types of seizures including infantile spasms which predominate in IESS [8]. Additionally, seizure burden differs between syndromes ranging from self-limiting seizures (e.g. SeLNE and SeLECTS) that abort spontaneously at a certain age to frequent uncontrolled seizures in severe syndromes like Lennox–Gastaut syndrome (8).

**Table 1:** Syndromic epilepsies at different age groups and possible associated neurodevelopmental co-morbidities and monogenic aetiologies

Epilepsy Syndrome^¥^	Abbreviation	Incidence rates^#^	Age of onset	Neurodevelopment	Monogenic aetiology	Implicated genes
Neonatal/infantile onset						
Early infantile developmental and epileptic encephalopathy (Ohtahara Syndrome)	**EIDEE**	10	1 w-3 months	Severely abnormal	Yes	*KCNQ2*,* STXBP1*,* CDKL5*,* SCN2A*,* SLC25A22*,* ARX*,* SPTAN1*,* KCNT1*,* SEPSECS*,* ERBB4*,* SIK1*,* SLC25A22*,* PIGA*,* PIGQ*,* GABRB2*,* SETBP1*,* STXBP1*,* GNAO1*,* DOCK7*,* SCN8A*,* UBA5*
Epilepsy of infancy with migrating focal seizures	**EIMFS**	N/A	months- 1 year	Severely abnormal	Yes in 70%	KCNT1^*^, *KCNQ2*,* SCN1A*,* SCN2A*,* PLCB1*,* TBC1D24*,* CHD2*,* QARS*,* SCN8A*,* SLC25A22*,* SLC12A5*,* ITPA*,* ATP1A3*
Infantile epileptic spasms syndrome (West syndrome)	**IESS**	30	1–24 months	Abnormal	Yes in 41% ‡	*ARX*,* CDKL5*,* STXBP1*,* IQSEC2*,* TSC1*,* TSC2*
Self-limited Familial neonatal epilepsy	**SeLNE**	5.3	1 day − 1 month	Normal	Yes	*KCNQ2*,* KCNQ3*,* SCN2A*
Self-limited Familial infantile epilepsy	**SeLIE**	14.2	1 month − 1 year	Normal	Yes	PRRT2^*^, *SCN2A*,* KCNQ2*,* KCNQ3*
Self-limited familial neonatal-infantile epilepsy	**SeLFNIE**	N/A	1 day-23 months	Normal	Yes	*SCN2A*,* KCNQ2*
Genetic epilepsy with febrile seizure plus	**GEFS+**	N/A	1 month − 1 year	Normal	Yes	*SCN1A*,* SCN1B*,* STX1B*,* HCN1*
Myoclonic epilepsy of infancy	**MEI**	N/A	months- 1 year	Normal^*^	No	
Gelastic seizures with hypothalamic Hamartoma	**GS-HH**	0.5	1 week-1 year	Abnormal	Yes in 5%	*GL13*
Sturge–Weber syndrome	**SWS**	2–5	1–12 months	Abnormal	Yes (somatic)	*GNAQ*
Dravet syndrome	**DS**	6.5	1–20 months	Abnormal	Yes	SCN1A^*^, *GABRG2*,* GABRA1*,* STXBP1*
Glucose transporter 1 deficiency syndrome	**GLUT1DS**	4.2	1–12 months	Abnormal	Yes	*SLC2A1*
PCDH19 clustering epilepsy	**PCDH19-CE**	2.4	1–60 months	Abnormal	Yes	*PCDH19*
Childhood onset						
Self-limited epilepsy with centrotemporal spikes	**SeLECTS/Rolandic**	6.1	2-12years	Normal^*^	Rarely yes	*GRIN2A*,* FMR1*
Self-limited epilepsy with autonomic seizures	**SeLEAS**	N/A	2-12years	Normal	Rarely yes	*SCN1A*
Childhood occipital visual epilepsy	**COVE**	N/A	2-12years	Normal^*^	No	
Photosensitive occipital lobe epilepsy	**POLE**	10–50	2-12years	Normal	No	
Febrile infection-related epilepsy syndrome	**FIRES**	0.1	2-12years	Abnormal	No	
Hemiconvulsion-Hemiplegia-Epilepsy	**HHE**	N/A	1 month- 5 years	Abnormal	No	
Lennox–Gastaut syndrome	**LGS**	10–50	2-12years	Severely abnormal^*^	No‡	
DEE with spike-and-wave activation in sleep	**DEE-SWAS**		2-12years	Abnormal	Yes&	*GRIN2A*
EE with spike-and-wave activation in sleep (Landau–Kleffner syndrome)	**EE-SWAS**		2-12years	Abnormal	Yes&	*GRIN2A*
Genetic Generalized epilepsies						
Epilepsy with myoclonic absences	**EMA**	N/A	2-12years	Abnormal in 50%	Yes in 30%	*SLC2A1*
Epilepsy with eyelid myoclonia	**EEM**	N/A	2-12years	Abnormal &	Rarely	*CHD2*,* SYNGAP*,* NEXMIF*
Epilepsy with myoclonic–atonic seizures Doose Syndrome	**EMAtS**	10	2-12years	Abnormal	Yes &	*SCN1A*,* SCN2A*,* SCN1B*,* SLC2A1*,* CHD2*,* SLC6A1*,* SLC2A1. NEXMIF*
Idiopathic generalized epilepsies						
childhood absence epilepsy	**CAE**	8	2–13 years	Normal^*^	No	
Juvenile absence epilepsy	**JAE**	10	8–20 years	Normal^*^	No	
juvenile myoclonic epilepsy	**JME**	10–30	8–40 years	Normal^*^	No	
generalised tonic clonic seizures alone	**GTCA**	30	5–40 years	Normal^*^	No	
Variable age of onset						
Epilepsy with auditory features	**EAF**	N/A	10–30 years	Normal	Yes in 50%	*LGI1*,* RELN*,* MICAL1*
Familial focal epilepsy with variable foci	**FFEVF**	N/A	1 month- 52 years	Normal^*^	Yes	*TSC1*,* TSC2*,* DEPDC5*,* NPRL2*,* NPRL4*
Familial mesial temporal lobe epilepsy	**FMTLE**	N/A	3–63 years	Normal^*^	Rarely	
Mesial temporal lobe epilepsy with hippocampal sclerosis	**MTLE-HS**	3.1–3.4	Any age	Mildly abnormal	No	
Rasmussen syndrome	**RS**	0.017–0.024	1–10 years	Abnormal	No	
Sleep-related hyper-motor (hyperkinetic) epilepsy	**SHE**	1.8–1.9	2 month- 64 years	Normal^*^	Yes &	*CHRNA4*,* CHRNB2*,* CHRNA2*,* KCNT1*,* DEPDC5*,* NPRL2*,* NPRL3*,* PRIMA1*
Epilepsy with reading-induced seizures	**EwRIS**	N/A	10–46 years	Normal	No	
Progressive myoclonus epilepsies	**PME**		2–50 years	Abnormal	Yes	*TPP1*,* CLN3*,* CLN6*,* CTSD*,* PPT1*,* POLG*
Lafora type	**PME-Lafora**	0.1–0.9	6–19 years	Abnormal	Yes	*EPM2A* and *EPM2B*
Unverricht–Lundborg type	**PME-ULD**	N/A	7–13 years	Mildly abnormal	Yes	*EMP1* (repeat expansion)

*(¥) The information presented in this table are based on 3 publications by the international league against epilepsy*: [8–10]

*(#) per 100*,*000 live births*

(*) in most cases,* (&) in some cases*,* (N/A) not available*

*‡ Chromosomal abnormalities and Copy number variants are widely reported so chromosomal microarray should be utilized*

Another differential feature in epilepsy diagnosis is aetiology. Different acquired aetiologies, including brain lesions, stroke, or central nervous system infections, can cause epilepsy [12]. To exemplify, mesial temporal lobe epilepsy (MTLE) is mostly attributed to early brain damage or infection [13]. However, 70 to 80% of epilepsy cases are not attributed to these factors and are termed genetic or idiopathic epilepsy [12, 14]. Common types of epilepsy including MTLE are predominantly associated with polygenic risk factors that increase carrier susceptibility [15, 16]. Monogenic epilepsy is relatively rare, manifesting in infancy or early childhood and usually associated with developmental delay, cognitive impairment, or other neurological abnormalities [15–17]. The incidence of epilepsies caused by genetic variations was estimated to be around 1 per 1000 live births in a prospective population-based study in Scotland [7]. Monogenic aetiologies explain about 20–40% of recruited cases with early-onset epilepsies in different studies [18–21]. Several established epilepsy syndromes are associated with monogenic causes (Table 1). Moreover, phenotypes that have specific electroclinical features repetitively observed in affected individuals and are associated with single-gene aetiologies are classified into aetiology-based syndromes, e.g. *PCDH19* clustering epilepsy and *GLUT1* deficiencies, or *KCNQ2*-DEE [8].

## Genetics of monogenic epilepsy

Neuronal networks are complex, and different proteins are involved in the same pathological mechanism, leading to heterogeneity in epilepsy associated genes. Among these, more than 400 new genes have been identified over the past 6 years (2017–2023) as being associated with epilepsy due to advancements in sequencing technologies [22]. However, asserting gene-disease linkage requires careful analysis of all clinical and experimental evidence to avoid false-positive results that misguide the epilepsy literature and, more importantly, delay proper diagnosis and treatment in affected patients [23]. Therefore, Global initiatives continue to refine the catalogue of genuine epilepsy-associated genes through systematic evaluation of genetic evidence [22, 24, 25]. The Clinical Genome Resource (ClinGen) has been instrumental in this process, introducing a semiquantitative framework that classifies gene–disease relationships into six levels of evidence—refuted, disputed, limited, moderate, strong, and definitive—based on clinical and experimental data [23]. As of September 2025, the ClinGen’s Epilepsy Gene Curation Expert Panel had reviewed 142 genes, identifying 9 refuted, 6 disputed, and 95 definitive gene–phenotype associations (**Online Resource 1**) [26]. Associations are typically refuted when variants occur at high population frequencies, alternative mechanisms are identified, segregation is unsupported, or case–control studies show no enrichment [22, 23, 26].

Several other groups have developed epilepsy gene panels using resources such as OMIM, HGMD, ClinVar, and curated literature (**Online Resource 2**). The Genes4Epilepsy consortium integrates data from ClinGen, PanelApp Australia, and PubMed into a publicly available, continuously updated database containing 1,052 genes, most linked to developmental and epileptic encephalopathies (DEE) (as of September 2025; github.com/bahlolab/genes4epilepsy) [24]. Similarly, Gracie et al. developed the Seizure-Associated Genes Across Species (SAGAS) database, listing 2,876 genes, including 2,404 with at least one line of evidence for association with human epilepsy and 154 classified as high-confidence epilepsy genes based on human and animal data [25].

Most confirmed epilepsy genes are also implicated in neurodevelopmental disorders, with clinical presentations ranging from mild developmental delay to severe DEE. In agreement, DEE-associated genes display the highest degree of differential gene expression during development stages compared to other epilepsy genes, restressing their role in neurodevelopment [27]. In some syndromes, developmental encephalopathy predominates, with epilepsy as a variable feature. Therefore, Zhang et al. classified epilepsy genes into three major categories: core epilepsy genes (*n* = 168), genes associated with neurodevelopmental or structural brain abnormalities (*n* = 364), and genes linked to multisystem syndromic disorders (*n* = 974) [22]. Their analysis, integrating Genes4Epilepsy, SAGAS, OMIM, HGMD, and PubMed, also identified the top 100 genes most frequently mutated in epilepsy, based on HGMD and the China Epilepsy Gene 1.0 Project [22].

To improve diagnostic accuracy, the Ontario Epilepsy Genetics Testing Program (OEGTP) established a curated panel of 190 genes with strong evidence for epilepsy association, aiming to minimize inconclusive test results [28]. Diagnostic laboratories now offer a range of targeted sequencing options, from focused panels covering a few hundred genes to comprehensive ones exceeding a thousand. For example, GeneDx provides the Comprehensive Epilepsy Panel (144 genes) and the EpiXpand Panel (1,741 genes) (**Online Resource 3**).

**Fig. 1:**
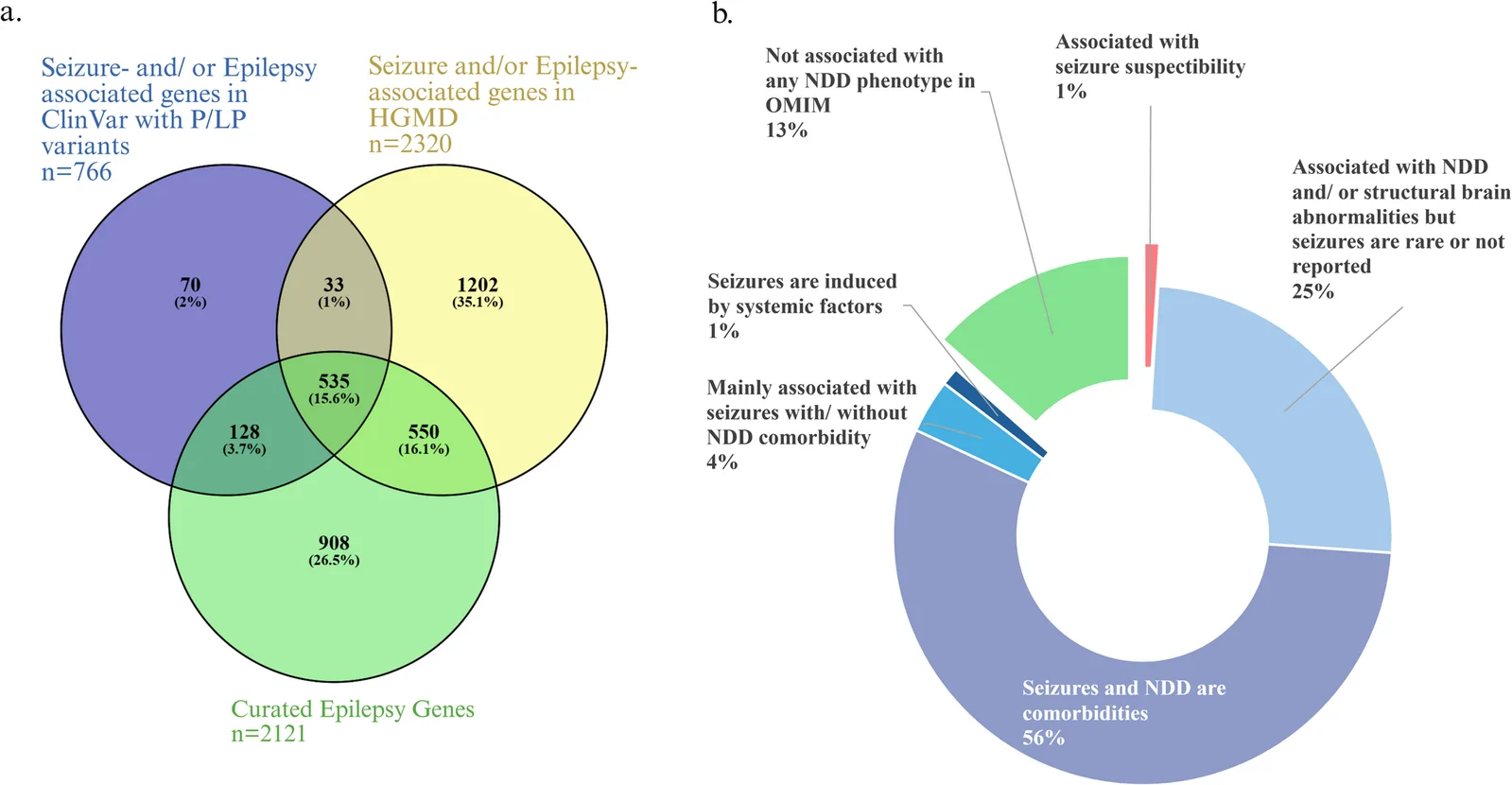
Distribution of the curated epilepsy genes (*n* = 2121) in databases. (**a**) A Venn diagram depicting the fractions of shared and non-shared genes between the curated epilepsy genes in this study (*n* = 2121), Seizure- and/or epilepsy-associated genes in HGMD (*n* = 2320) and in ClinVar (*n* = 766) with pathogenic or likely pathogenic variants. (**b**) A doughnut chart summarizing OMIM phenotypes associated with the curated epilepsy genes. Most genes are associated with both seizures and neurodevelopmental disorders (NDD). 11% of genes are not associated with epilepsy nor any neurodevelopmental disorder (NDD) in OMIM. Few genes are associated with susceptibility (risk) to certain phenotypes that have seizures in their clinical synopsis in OMIM. In 1% of the genes, phenotypes exhibit seizures induced by systemic factors including hypoglycaemic, hypomagnesemia, hypocalcaemia, elevated serum bilirubin or jaundice and hyper- or hypotension. The data presented in this figure is based on the curated epilepsy gene list, HGMD and ClinVar gene list presented in the Online Resource 2 and 4. The Venn diagram was constructed through Venny 2.1 online platform (Oliveros, J.C. (2007–2015) Venny. An interactive tool for comparing lists with Venn’s diagrams. https://bioinfogp.cnb.csic.es/tools/venny/index.html)

In the present study, we compiled and annotated a list of epilepsy-associated genes from three main sources, genes reported in targeted gene panels in 6 diagnostic laboratories (GeneDx, Blueprint, Centogen, Prevention, Flugent and Invitae), genes associated with seizure and/or epilepsy in OMIM, as well as genes listed in the ClinGen’s Epilepsy Gene Curation Expert Panel, OEGTP, Genomics England (Version 7: Early-onset or syndromic epilepsy), and 2 literature sources (**Online Resource 1–3**). After removing all duplicates, the genes listed as refuted or disputed by ClinGen’s Panel (*n* = 8) was removed to have a final dataset of 2121 curated epilepsy genes which are annotated in **Online Resource 4**. These curated epilepsy genes significantly overlap with genes associated with seizure and/or epilepsy in HGMD (1085 out of 2121 genes; 51%) and with seizure and/or epilepsy-associated genes having pathogenic or likely pathogenic variants in ClinVar (663 out of 2121 genes, 31%) (Fig. 1a). Most of the curated epilepsy genes are associated with NDD comorbidity in addition to seizure or epilepsy in OMIM, emphasizing the shared genetic basis of epilepsy and NDDs (Fig. 1b). Nevertheless, many genes are solely linked to NDD, or neuropsychiatric disorders and seizures are rarely reported or totally absent in the clinical synopsis in OMIM. Noteworthy, several genes that appear in clinical epilepsy reports in HGMD remain unlisted as epilepsy-related genes in OMIM, suggesting possible under-recognition of their epileptic associations or weak evidence of pathogenicity in these clinical reports (Fig. 1). Reclassification rates of ClinVar’s pathogenic or likely pathogenic variants are much higher than HGMD’s disease-causing mutations (DM and DM?) contributing to lower rates of false-positive data in inborn error of metabolism phenotypes in ClinVar compared to HGMD [29].

### Pathogenesis mechanisms of monogenic epilepsy

**Fig. 2:**
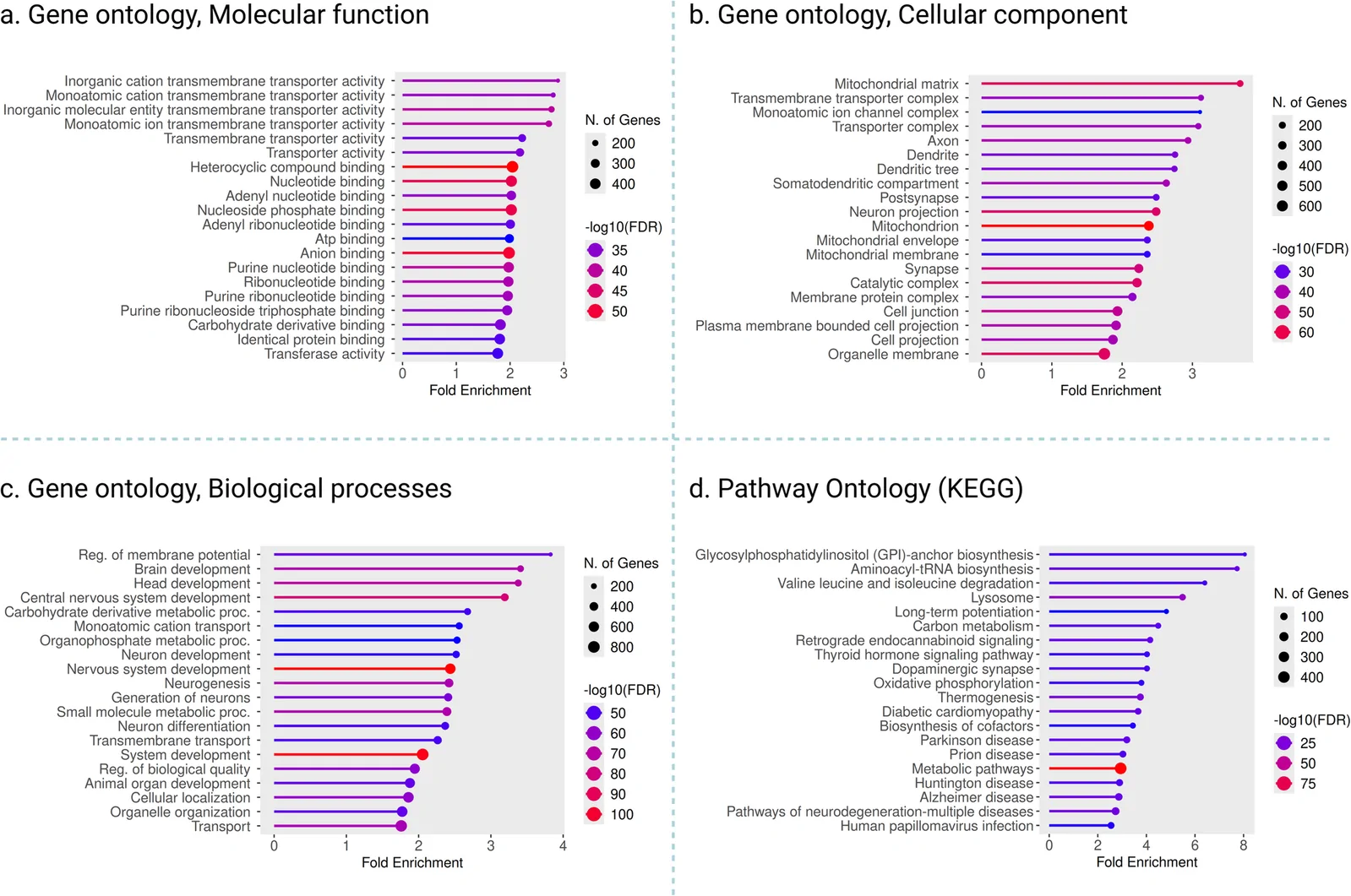
Enrichment analysis of ontologies associated with the curated epilepsy genes (*n* = 2121). a., b. and c. represent the molecular function, cellular components and biological process categories of gene ontologies. d. Pathway ontology of these genes based on the Kyoto Encyclopaedia of Genes and Genomes (KEGG). All the graphs, Y-axis lines display the top 20 enriched ontology terms. The X-axis shows the fold enrichment. Fold enrichment calculates how genes in each ontology is enriched with the provided gene list (*n* = 2121) compared to a background of reference genes. The size of the end bubble in each ontology line reflects the number of genes that overlap with the genes in that ontology. The color of the lines represents a measure of the false discovery rate (FDR) or possible false positive results in the enrichment analysis in a range from red (low FDR) to blue (high FDR). These figures are extracted from ShinyGO (0.85 version) as output of provided epilepsy gene list choosing Human in species section Reg. = Regulation

Enrichment analysis of the curated epilepsy genes revealed involvement of these loci in divergent cellular and neuronal pathways (Fig. 2). At the molecular level, genes encoding subunits of voltage- and ligand-gated ion channel are highly enriched (Fig. 2a). In agreement, regulators of membrane potentials and ion channel complex are well represented in the products of epilepsy genes at the cellular and biological levels (Fig. 2b and c). Genes encoding transporters of vitamins, nutrients and other substrates involved in cellular metabolism are additionally enriched epilepsy genes (Fig. 2a and c). Relatedly, significant proportion of epilepsy genes are involved in regulating metabolic pathways, lysosomal function, mitochondrial machineries, and other anabolic or catabolic mechanisms according to pathway and cellular ontologies (Fig. 2b and d). Intracellular signalling pathways guiding neuronal differentiation, proliferation and development are overrepresented in the biological process ontologies of epilepsy genes Fig. 2c. Most of the genes in the pathway of Glycosylphosphatidylinositol-anchor biosynthesis (22 out of 25 genes) overlap with the epilepsy gene panel leading to 10-fold enrichment of this pathway (Fig. 2d). GPI anchoring of proteins mediates various signalling mechanisms regulating neuronal development and function [30].

More broadly, genes involved in the biosynthesis of aminoacyl-tRNA and hence mRNA translation are overrepresented in the epilepsy loci (Fig. 2d). The wide diversity in epilepsy gene ontologies is reflective of complex interactions between intracellular pathways and neuronal networks. Abnormality in any component of this complex network result in excitatory-inhibitory neuronal imbalance that drives abnormal synchronized discharges observed in the electroencephalography during seizures [31, 32]. These networks are additionally involved in brain development and higher brain functions which reemphasize the fact the many epilepsy genes are associated with neurodevelopmental disorders (Fig. 1b**)**.

### Gene regulating neurotransmission

GABAergic and glutamergic neurotransmission governs the primary inhibitory and excitatory neurotransmission in the brain, respectively [32, 33]. Dysfunction in these neuronal systems leads to hyperexcitability and epilepsy [33–35]. Both gain-of-function (GOF) and loss-of-function (LOF) variations in genes encoding glutamate and GABA receptors have been reported to result in epilepsy or epileptic encephalopathies in different studies [36–39]. Other neurotransmitter systems also participate in the process of excitation-inhibition disturbance. Variants in nicotinic acetylcholine receptor subunit CHRNA2 have been reported to cause nocturnal frontal lobe epilepsy [40].

### Genes regulating voltage-gated ion channels

Glutamate and GABA are substrates of ligand-gated ion channels that alter the excitability of neurons and maintain excitation-inhibition balance. However, voltage-gated ion channels are the driving force of neuronal excitability (Fig. 2) [36]. These ion channels include voltage-gated sodium, calcium, and potassium and chloride channels, Hyperpolarization-activated cation channels, sodium leak channel, and Inward Rectifying Potassium Channels [36, 41]. LOF and GOF variants in genes encoding ion channels have been observed to result in a spectrum of epilepsy and neurodevelopmental phenotypes [36, 41–43].

### Genes regulating neuroinflammation

Microglia and astrocytes are “housekeeping” cells that regulate homeostasis, excitability, plasticity, neurogenesis, blood-brain barrier, and immune activity [44–46]. Additionally, astrocytes are the hub for glutamate/GABA-glutamine cycle which is essential for synaptic function [47]. Dysfunction in glial function and chronic neuroinflammation has been correlated with epileptogenesis in different epilepsy types [45, 46].

### Genes regulating synaptic function

**Fig. 3:**
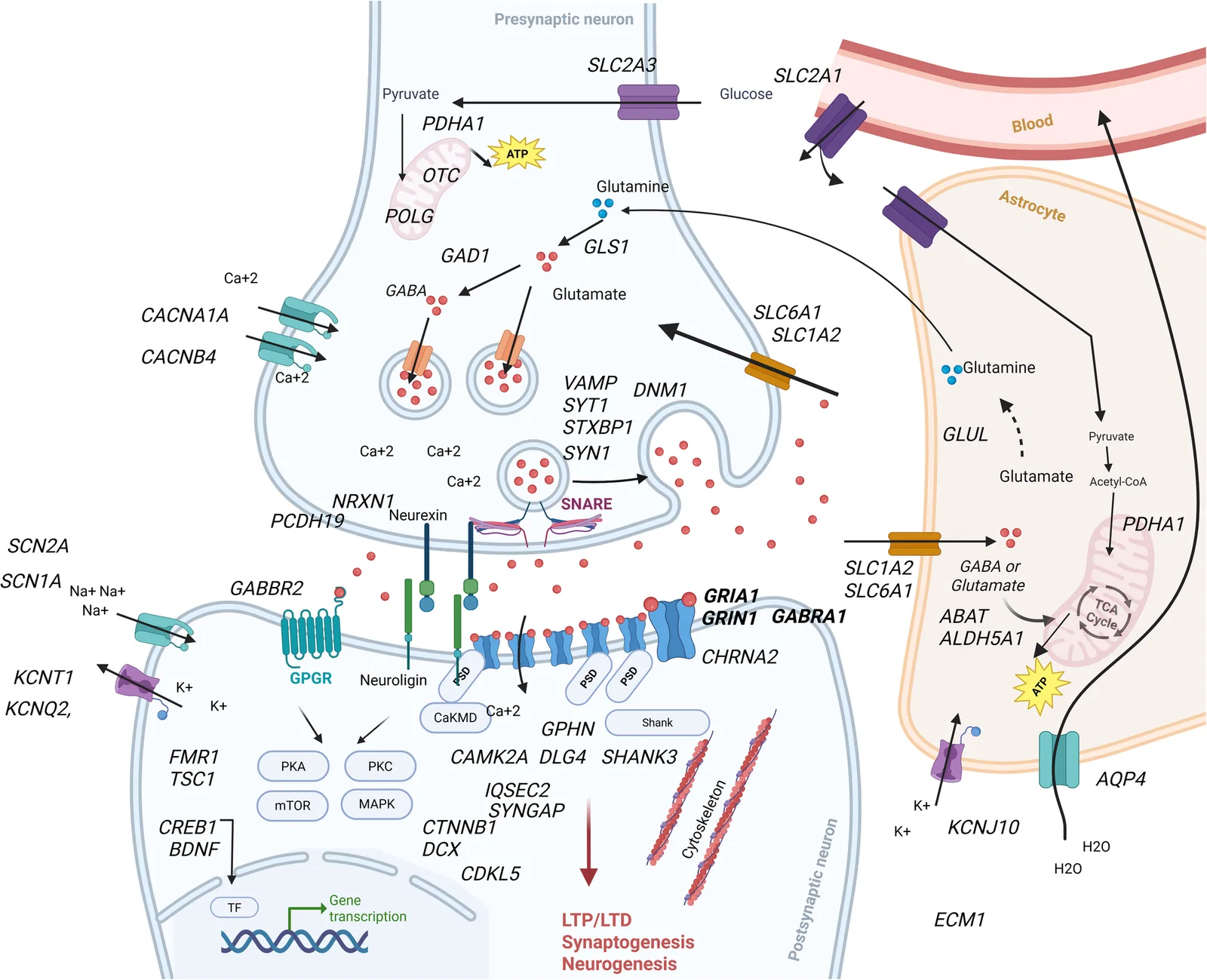
Visual summary of functions of epilepsy genes as illustrated by the distribution of their products across different components of the neuronal synapse. The gene names are presented in italic font next to the possible cellular location of their protein products. Starting from top to bottom: Glucose enter the CNS through the solute carrier (SLC2A1) via the blood-brain barrier maintained by astrocytes. Glucose is then transported to neurons via another carrier (SLC2A3). Glucose is transformed into pyruvate and then to acetyl-CoA via Pyruvate dehydrogenase complex subunits (including PDHA1). This complex additionally converts glutamate into α-ketoglutarate which is in addition to Acetyl-CoA is part of the mitochondrial citric acid (TCA) cycle that produce ATP. GABA participate in the TCA cycle in neurons and astrocytes through enzymes encoded by ABAT and ALDH5A1 genes. Astrocytes uptake glutamate and GABA from the synapse and pass them into the TCA cycle and convert glutamate into glutamine via glutamine ligase (GLUL) which is in turn transported to the presynaptic terminal. Glutamine can be converted to glutamate or GABA via enzymes encoded by GLS1 and GAD1 genes. Astrocytes maintain water homeostasis and neuronal excitability through the expression of aquaporins (AQP4) and inward-rectifying potassium channels (KCNJ10). Mitochondria have other enzymes that participate in cellular metabolism including urea cycle enzymes (e.g. OTC). The mitochondrial proteins are essential for energy production, and mtDNA is replicated and maintained by the POLG enzyme. Entry of calcium through CACN1A or CACNB4 channels trigger the release of synaptic vesicle contents through the SNARE complex pathway that includes VAMP, SYT1, DNM1 genes. Adhesion molecules (e.g. PCDH19 and NRXN1) maintain tight synaptic cleft and interact with postsynaptic machinery including postsynaptic density proteins (GPHN or DLG4) and SHANK3 scaffolding proteins to maintain stable synaptic transmission. Released neurotransmitters interact with their ligand-gated ion channels (e.g. GRIA1, GRIN1, CHRNA2 and GABRA1) or G-protein coupled receptors (e.g. GABBR2) to transmit their signal to postsynaptic neurons. Activation of these receptors can trigger subsequent short-term activation of postsynaptic voltage-gated ion channels. Additionally, cascades of intracellular signaling pathways can be activated including kinases (e.g. CDKL5) and transcription factors (e.g. CREB1) which are associated with a variety of long-term effects including long-term potentiation (LTP), long term depression (LTD), synaptogenesis and neurogenesis. Extracellular matrix protein 1 (ECM1) regulates the extracellular matrix which provides a structural support for the synapses GPCR: G-protein-coupled receptors, LTP/LTD: Long term potentiation/long term depression components of neuroplasticity. PSD: postsynaptic protein density TF: Transcription factors. The diagram was created in Biorender and extracted for publication purpose with license number: GS28V93WYI

Crosstalk between neuronal systems as well as glial cells is pivotal for the function of the central nervous system. Miscommunication between neurons result in different neurological abnormalities including autism and epilepsy [48]. Genes regulating presynaptic, axonal, dendritic, and postsynaptic activities are highly enriched in the curated epilepsy gene panel (Fig. 2B). Synapses display dense expression of many proteins involved in the regulation of chemical signal to achieve stable communications between neurons (Fig. 3).

Upstream of the synaptic junction, descending electrical currents activate VGCCs (e.g. *CACNA1A*, *CACNB4*) at the axonal terminal which in turn triggers calcium influx that sparks subsequent vesicle-mediated neurotransmitter release to the synaptic cleft (Fig. 3). Vesicles are tethered to the cytoskeleton by Synapsin I (*SYN1*) that, in association with the SNARE complex, orchestrates vesicular trafficking to the pre-synaptic membrane [49, 50]. SNARE complex includes Synaptotagmin-1 (*SYT1* gene), Syntaxin-1B (*STX1B* gene), Syntaxin-Binding Protein*1* (*STXBP1* gene), Synaptosomal-Associated Protein 25kDa (*SNAP25* gene), and Vesicle-associated membrane protein (*VAMP* gene) [51, 52]. Upon the release of vesicular content, the recycling of synaptic vesicles and vesicle availability for upcoming signals are governed by different proteins, including Dynamin-1 (*DNM1* gene) and the aforementioned Synapsin I protein [52]. At the postsynaptic level, several proteins play critical roles in regulating postsynaptic “architecture” that maintain effective and stable synaptic transmission [53]. These include adhesion molecules that tightens post-synaptic dendrites to pre-synaptic axonal terminals including neurexins (*NRXN* gene), neuroligins (*NGLN* gene) and calcium-dependent protocadherin (*PCDH19* gene) [54, 55]. Furthermore, scaffolding proteins like SH3 and Multiple Ankyrin Repeat Domains 3 (*SHANK3* gene), post-synaptic density protein-95 (*DLG4* gene) and Gephyrin (*GPHN* gene) anchor post-synaptic receptors to the cytoskeleton and intracellular proteins to maintain stable signalling (Fig. 3) [56–58]. Dysfunction in any component of these proteins result in synaptopathies which manifest in neurodevelopmental disorders and epilepsy [48].

### Genes regulating neuroplasticity

Activation of postsynaptic neurons triggers subsequent cascades of activations of different intracellular signalling machinery, including kinases (e.g. CaMKII, PI3K, and mTOR), growth and transcription factors (e.g. *BDNF*, *UBE3A*, *CREB1*, *MEF2*, β-catenin, and NPAS4) and epigenetic regulators (e.g. *HDAC*, *DNMT1*) (Fig. 3) [59–61]. In turn, complex interactions of these activated intracellular proteins promote adaptive, long-term functional and structural and changes in neuronal networks, a process known as plasticity. Functional plasticity is exemplified by long-term potentiation and Long-term depression of glutamate signals during memory formation [60, 62]. Structural plasticity extends to synaptic rewiring synaptogenesis, axonal growth, and neurogenesis [63]. Plasticity plays a crucial role in neuronal and synaptic maturation during brain development and in neurocognitive function in the adult brain [62]. Genetic variants in neuroplasticity networks lead to developmental abnormalities, structural brain malformations and possibly epilepsy (Fig. 2d) [64, 65]. Focal cortical dysplasia, grey matter heterotopia, and lissencephaly are common neuroanatomical defects in rare genetic epilepsies that are often pharmaco-resistant [66–69]. Association of each monogenic aetiology with cortical malformations and/or other abnormal structural changes detected in the magnetic resonance imagining (MRI) is addressed in the annotation of the curated epilepsy genes (Online Resource 1).

### Genes regulating cellular metabolism

Synthesis and degradation of substances are ongoing processes in active cells, which produce energy, regulate intracellular functions, remove waste, and recycle essential nutrients and building blocks. Excitable cells, including neurons, are metabolically active cells that require continuous energy and metabolic cycles. Elevated excitability and seizure activity alter the underlying metabolic activity of neurons [70]. Similarly, abnormalities of any component of cellular metabolism are expected to disturb neuronal excitability as well as glial function [70]. Genes implicated in cellular metabolism include those regulating mitochondrial, lysosomal, peroxisomal, and other metabolic systems, as well as genes encoding transporters of nutrients and metabolites (Fig. 2a and c). Variants in genes regulating metabolic pathways result in inborn errors of metabolism that are associated with neurodevelopmental disorders and high risk of early-onset epilepsy [70–72].

## Phenotypic pleiotropy in monogenic epilepsy

Besides locus heterogeneity, another complexity of analysis and diagnosis of genetic disorders is phenotypic heterogeneity where pathogenic variants within the same gene do not invariably produce the same clinical phenotype. In some genetic disorders, presence of the pathogenic variants may not produce any phenotype in some individuals, i.e. incomplete penetrance. In some genetic disorders, variability in expressivity of variants in a gene result in a spectrum or continuum ranging from mild to severe phenotypes [73]. For example, genetic variations in *SCN1A* gene produce a phenotypic continuum from mild genetic epilepsy with febrile seizure plus (GEFS+) to severe DEE [74]. Different factors play a role in phenotypic heterogeneity.

### Allelic heterogeneity: Variability in the causative variants

The leading cause of phenotypic heterogeneity in genetic disorders is the effect of location and type of variants on the resultant phenotypes. Both LOF and GOF variants in *SCN1A* gene result in epilepsy phenotypes. Early onset epilepsy was found to be associated with GOF, and delayed onset after 3 months of age was seen with LOF variations [75]. Furthermore, truncating variants in *SCN1A* gene were associated with more severe presentations of DS compared to missense variants [74, 76]. Missense variants in non-conserved areas of *SCN1A* gene may trigger late-onset GEFS+, in contrast to missense variants in functionally critical regions like the N-terminus or channel pore sequence that result in severe early-onset Dravet syndrome [74, 76, 77]. Missense variant in functionally important areas may result in LOF akin to truncating variants [74]. Canonical splice site variations induce more severe functional protein damage and phenotypes than deep intronic variants, which have pathogenic implications for splicing [78]. Additionally, non-coding intronic or promoter region variants can alter gene expression [79]. A rare variant in the promoter region of *SCN1A* resulted in reduced gene expression. It was suspected to explain the mild phenotype and incomplete penetrance in a patient with partial epilepsy and febrile seizures [80]. Structural variants, such as copy number variations, can alter gene dosage or disrupt multiple genes, resulting in distinct clinical presentations compared to monogenic disorders [17].

### Polygenic interactions and minimal individual polymorphism effects

Common polymorphisms are not pathogenic on their own but can interact with pathogenic variants to alter phenotypic presentation. There are many studies that analyse the contribution of these risk polymorphisms in the development of polygenic epilepsy. Contribution of each common polymorphism can be minimal compared to a background of many polymorphisms (polygenic risk scores; PRSs) interacting together to increase the risk of developing or modifying a phenotype [16]. Very recently, common polymorphism in 26 known epilepsy genes have been observed to be uniquely elevated in 29,000 patients with different phenotypes of epilepsy compared to controls [81]. These 26 genes are primarily involved in ion channels and neurotransmission. Higher burden of these polymorphisms (i.e., higher polygenic risk scores) was observed in patients with familial epilepsies compared to their unaffected relatives and controls [82]. These findings of PRS predicting epilepsy were supported by a recent meta-analysis of 11 studies with patients of European ancestry [83]. Each seizure type (focal, generalized or mixed) was found to have unique PRS in multiplex families [84]. These findings can be theoretically extended to explain phenotypic heterogeneity in monogenic epilepsies [84]. Within families with monogenic GEFS+ syndrome, PRS proportionally correlates with the severity of phenotypes [85]. In two unrelated probands having the same splicing variant in *SCN1A*, lower epilepsy-related PRS and higher intelligence-related PRS were found in the patient with milder Dravet syndrome [86]. Noteworthy, family-based genetic analysis enables a reliable examination of haplotype-based interactions between common polymorphisms and causal variants [87].

### Epistatic variant interactions and phenotypic diversity

The main pathogenic variant can interact (epistasis) with a second variation in the same gene or other epilepsy genes in the same pathway resulting in phenotypic heterogeneity [88]. To illustrate, unaffected carriers shared a second modifier variant in the *SCN1A* gene that reversed the pathogenicity of the pathogenic variant in a family with an *SCN1A*-related disorder [85]. Furthermore, variants in the promoter regions of the wild-type allele of the *SCN1A* gene may result in reduced levels of the normal copy of the VGSCs, thereby worsening haploinsufficiency and leading to a more severe phenotype [89]. In various scenarios, a mixed phenotype arises from the combined effect of two independent disease-causing variants in different genes [90]. For example, cases having Dravet syndrome with frontal cortical dysplasia can be explained by the presence two pathogenic variants in *SCN1A* and *DEPDC5* genes [86]. Multiple rare and deleterious variants form a variant load or burden the found to alter the phenotype of the main causative variant. Burden of rare variants was found to augment the risk of sudden unexpected death in epilepsy [91]. Variant load was found to vary among different types of epileptic lesions resected from patients with epilepsy [92].

### Heterogeneity of underlying molecular mechanisms

Some epilepsy genes are associated with different phenotypes with more than one pattern of inheritance. These phenotypes can be distinct or in a continuum from mild phenotype induced by monoallelic variants to severe phenotypes caused by biallelic variants [23]. Haploinsufficiency of the *SCN1A* gene may not be the only mechanism of *SCN1A*-related phenotypes; mutated alleles may negatively influence wild-type alleles in a negative dominance mechanism, and GOF variants augment the activity of sodium ion channels, causing modified phenotypes [78]. Another complicated molecular mechanism is mosaicism resulting from de novo variants affecting only a fraction of cells. Mosaicism may lessen the severity or penetrance of the phenotype as observed in *SCN1A*-related phenotypes [74, 93]. Mosaicism can be produced by imprinting or epigenetic factors observed in *PCDH19*-related phenotypes [94, 95]. Mosaicism is one proposed mechanism of pathogenesis in these phenotypes [54, 94, 95]. This proposed mechanism was supported by clinical and experimental studies showing that the disease is expressed in mosaic carrier females (due to X-inactivation) but not in non-mosaic carrier males with only one copy of X chromosome [54, 94].

## Genetic testing in epilepsy

Genetic testing in epilepsy can reveal genetic aetiology in about 22% to 40% of cases [96, 97]. The predictors of high diagnostic yields that can be inferred from many studies help in informing indications for genetic testing in epilepsy patients. The diagnostic yields are influenced by patient- and assay-related factors. It is more likely to find a single-gene aetiology in cases with early-onset epilepsy, relevant family history, comorbid neurodevelopmental abnormalities and/or poor drug response [98–102]. Parental consanguinity is a predictor of high diagnostic yields in autosomal recessive epilepsy [103–106]. Around 60% of the curated epilepsy genes are associated with rare phenotypes, autosomal recessive inheritance (**Online Resource 4**). Nevertheless, dominant inheritance and de novo variations are common in epilepsies, particularly DEEs [24]. Diagnostic rates can go above 50% in cases with DEE, reaching up to 64% in studies recruiting patients with neurodevelopmental abnormalities, particularly intellectual disabilities [96]. Although genetic testing is recommended in early childhood to implement possible therapeutic measures that rescue the developing brain, identifying genetic aetiology during adulthood is additionally beneficial in unexplained cases with neurodevelopmental comorbidities, early-onset seizures, and/or structural brain abnormalities [107–109]. Furthermore, the genetic field is constantly growing with new genes, defined pathogenic variants, and gene-disease associations which aid in the re-diagnosis of adult cases with previously inconclusive findings.

Choice of genetic test certainly influence diagnostic yields. Target gene panel sequencing (TGPS) is more cost-effective and provide better coverage in the targeted genes than exome sequencing (ES) or Genome sequencing (GS) [110]. Nevertheless, GS and ES outperform TGPS in finding causal variants [110–113]. TGPS test selection is based on empirically diagnosed or suspected phenotype, which may not address extensive phenotype heterogeneity and overlap in some genetic disorders including epilepsy [114–116]. ES and GS are considered “genotype-based” approaches to finding causal variants and should be more practical in the molecular diagnosis of heterogeneous disorders, including epilepsy [115]. Reanalysis of negative or inconclusive sequencing data is recommended every 1 to 2 years [75, 113–115, 117–119]. These next generation sequencing (NGS) techniques certainly outperform conventical techniques including single-gene sequencing [110, 114]. Having said that, high costs, inadequate insurance coverage, and a lack of genetic expertise among healthcare providers may limit the implementation of genetic testing for highly susceptible individuals [120, 121]. 

## Management of epilepsy

The main aim of therapy is to reduce and possibly stop the recurrence of seizures. Frequency of seizures is one of the main criteria to follow the prognosis and treatment response in epilepsy [122]. Pharmacological treatment with antiseizure medications (ASM) is the first line approach in the management of epilepsy. There are many approved ASMs, including first-generation or conventional drugs (e.g. Valproate) and new agents designed to have higher selectivity and potency (e.g. Levetiracetam) (Table 2). These medications target molecular pathways involved in epileptogenesis mainly voltage-gated ion channels and GABA neurotransmission (Table 2). Despite appropriate use of ASMs, about 30% ofepilepsy cases display pharmacoresistance [123–126]. Pharmacoresistance or intractability is defined by seizure recurrence more than every 12 months despite two “trials” of appropriately chosen ASMs [124]. Notably, pseudo-intractability like misdiagnosis, improper ASM dosing, or poor compliance should not be mistakenly reported as intractable epilepsy [126]. Several risk factors contribute to pharmacoresistance to ASM including the presence of one or more of the following based on several studies: very early onset, focal onset, high seizure frequency at onset, status epileptic at onset, structural abnormalities, and abnormal EEG [99–132]. The comorbidity of neurodevelopmental abnormalities in epilepsy increases the risk of intractability [128, 131].

**Table 2:** Antiseizure medication classes, indications, targets, metabolizing enzymes and transporters

ASM	Target gene (DrugBank)	Monotherapy use	Adjuvant use	Contraindicated	Genes encoding metabolizing Enzymes/transporters
Benzodiazepines					
Clobazam	*GABRA1*	Tonic-atonic seizures^#^	DS, EMAtS, LGS, Generalized seizures		*ABCB1*,* CYP3A4*,* CYP3A5*,* CYP2C18 CYP2C8*,* CYP2D6*,* CYP1A2*,* CYP2C19*,* CYP2C18*
Diazepam	*GABRA1*	Status epilepticus, emergency			
Lorazepam	*GABRA1*	Status epilepticus, emergency			
Clonazepam	*GABRA1*		Myoclonic seizures	Mixed types	*ABCC2*,* CYP3A4*,
GABA-R modulators					
Phenobarbital	*GABRA1*	Neonatal seizures	Generalized and focal seizures (any age)		*CYP2C9*,* CYP2C18*,* ABCB1*,* CYP2C19*,* CYP2C18*,* CYP2E1*,* CYP3A4*
Primidone	*GABRA1*, and other GABA-R subunits		Generalized seizures (any age)		
Stiripentol	*GABRA1-6*,* LDHA*,* LDHB*				
Ganaxolone	*GABRA1*	CDKL5 deficiency syndrome			
Vigabatrin	*ABAT*	Tuber sclerosis Complex	Focal seizures	Absence, myoclonic seizures, DS, tonic seizures, atonic seizures, LGS, EMAtS	
GABA transporter inhibitor					
Tiagabine	*SLC6A1*		Focal for patients ≥ 12 years	Absence, myoclonic seizures, DS, tonic, atonic seizures, LGS	*CYP2D6*,* CYP1A2*,* CYP1A1*,* CYP2C19*,* CYP3A4*
Glutamate blockers					
Felbamate	*GRIN2B & GRIN2A*		Tonic-clonic seizures, LGS		*CYP2E1*,* CYP3A4*
Perampanel	*GRIA1-4*		Generalized and focal seizures for patients ≥ 4 years		*CYP2B6*,* CYP1A2*,* CYP3A5*,* CYP3A4*
Glutamate release inhibitors					
Pregabalin	*CACNA2D1*		Focal seizures	Absence, myoclonic seizures, DS, Tonic seizures, atonic seizures, LGS, EMAtS	*SLC7A5*
Gabapentin	*CACNA2D1*	Focal seizures for patients for patients ≥ 12 years	Focal seizures for patients ≥ 6 years	Absence, myoclonic seizures, DS, tonic seizures, atonic seizures, LGS	*ABCB1*,* SLC7A5*
Synaptic release regulators					
Levetiracetam	*SV2A*,* CACNA1B*	Generalized and focal seizures, EMAtS for patients ≥ 16 years Absence, Infantile spasms, status epilepticus ≥ 16 years^#^	Focal seizures and DS for patients ≥ 1 month		*ABCB1*
Brivaracetam	*SV2A*		Generalized, focal, and myoclonic seizures for patients ≥ 4 years		*CYP2B6*,* CYP2C19*,* CYP2C9*,* CYP3A4*
Mixed effects					
Valproic acid	*HDAC9*, other targets	Generalized seizures, EMAtS, DS, LGS, tonic, atonic seizures (any age) Absence, Infantile spasms, status epilepticus (any age) ^#^	Focal (any age)	GLUT1 deficiency	*ABCB1*,* ABCC2*,* UGT2B7*,* UGT1A6*,* CYP2B6*,* CYP2A6*,* CYP2C19*,* CYP2C9*
Sodium channel blockers					
Cenobamate	*SCN1A*		Focal seizures		*CYP3A4*,* CYP2C19 CYP2E1*,* CYP2A6*,* CYP2B6*,* UGT2B7*,* UGT2B4*,* CYP2C19*
Lamotrigine	*SCN1A*,* CACNA1E*	Generalized and Focal seizures for patients ≥ 13 years, Absence, LGS^#^	Focal EMA for patients ≥ 2 years	DS, LGS, myoclonic seizures	*ABCB1*,* ABCG2*,* SLC22A1*,* UGT1A4*
Topiramate	*SCN1A*,* CACNA1C*,* GABRA1*,* GRIK1*	Focal seizures for patients ≥ 6 years Infantile spasms^#^	Focal and generalized seizures, DS, tonic seizures, atonic seizures, LGS for patients ≥ 2 years		*ABCB1*
Phenytoin	*SCN1A*,* SCN2A*,* SCN5A*,* NR1I2*,* KCNH2*,* CACNA1C*	Status epilepticus (any age) ^#^	Focal (any age)	Absence, myoclonic seizures, EMAtS	*ABCB1*,* ABCC2*,* ABCC1*,* COMT*,* CYP2B6*,* CYP2A6*,* CYP2D6*,* CYP2C19*,* CYP2C9*,* CYP2C8*,* CYP2E1*,* CYP3A4*
Carbamazepine	*SCN1A*	Focal seizures (any age) ^#^	Focal seizures at any age	Absence, myoclonic seizures, DS, tonic seizures, atonic seizures, LGS, EMAtS	*CYP3A4*,* EPHX1*,* ABCB1*
Oxacarbazepine	*SCN1A*, other subunits	Focal seizures for patients ≥ 6 years^#^		Absence, myoclonic seizures, DS, tonic, atonic seizures, LGS, EMAtS	*ABCB1*,* ABCC2*,* UGT2B7*,
Eslicarbazepine	*SCN11A*,* P2RX4*		focal seizures > 6 years		
Rufinamide	*SCN9A*,* GRM5*	Tonic seizures, atonic seizures, LGS			
Lacosamide	*SCN11A?*		Generalized and focal seizures for patients ≥ 4 years	DS, LGS	
T-type calcium channel inhibitors					
Ethosuximide	*CACNA1G*	Absence, EMAtS		Mixed types	*CYP2E1*,* CYP3A4*
Zonisamide	*CACNA1G*,* CACNA1H*,* CACNA1I*		Generalized, focal, myoclonic seizures for patients ≥ 6 years		*CYP3A4*

*DS: Dravet syndrome*,* EMAtS: Epilepsy with myoclonic–atonic seizures*,* LGS: Lennox–Gastaut syndrome*,

*Drug Targets*,* metabolizing enzymes and transporters are extracted from Drug Bank*,* and Guidelines for the proper use of ASMs are additionally displayed based on publicly available guidelines*

*namely: the National Institute for Health and Care Excellence and the European Medicines Agency guidelines.*

*# Second-line agent*

### Genetics role in pharmacoresistance

Genetic variations can alter drug response in several mechanisms. Firstly, drug resistance can arise due to changes that alter the activity and expression of proteins involved in the absorption, metabolism, or elimination of ASMs (Table 2) [125, 133, 134]. Polymorphism or pathogenic variations in these proteins alter drug pharmacokinetics, leading to reduced efficacy and/or tolerability. For example, polymorphism in the *ABCB1* transporter gene has been reported in drug-resistant epilepsies and was identified as a predictor of intractability [129, 135]. Reduced activity of the CYP2C9 enzyme due to SNPs predisposed to phenytoin resistance in epilepsy patients [136]. Secondly, pathogenic and polymorphic variations in drug target genes can lead to the development of drug resistance. For example, a specific combination of SNPs in the *SCN2A* gene resulted in altered response to valproate [137]. Variants in the *SCN1A* gene were linked with resistance to several SCBs and Valproate [138]. Furthermore, polymorphisms in *GABRA1*, *FAM131B*, and *CACNG5* genes were associated with resistance to VPA or CBZ in a cohort of 250 paediatric patients in Jordan [139]. Thirdly, complex brain networks and epileptogenesis processes contribute to poor drug response [125, 132, 134]. Therefore, ASMs may correct some pathways but not all epileptogenic processes [134]. Relatedly, epilepsies associated with enzyme deficiency are complex to treat without tackling the issue of missing enzymes or vitamins [71, 140]. Additionally, structural lesions caused by genetic defects (e.g. cortical malformation) may create an epileptogenic focus that is hard to overcome with only pharmacological treatment, requiring surgical correction [132, 134]. Noteworthy, resistance to ASMs brings about further resistance; uncontrolled recurrent seizures, particularly in the young brain, induce neuronal death, neurogenesis, and network changes like hippocampal sclerosis that require surgical correction [132]. Early molecular diagnosis is critical in cases with available intervention measures to overcome drug resistance. Actionable genes with available management measures should be prioritized for testing.

### Aetiology-based choice of antiseizure medications

In refractory epilepsy, treatment approach can be based on empirical evidence based clinical experience and treatment guidelines (Table 3). For example, monotherapy with valproate or lamotrigine in combination with adjuvant ASMs like Stiripentol, Cannabidiol, fenfluramine, or Clobazam is recommended for drug resistant DS and LGS syndromes [141]. Rufinamide is a third-generation sodium channel blocker (SCB) that is specifically indicated in refractory LGS [141]. This approach may improve seizure control, but they don’t tackle underlying aetiology. Some recommendations exist in the literature or guidelines for prioritizing ASMs based on the genetic aetiology. To elucidate, SCBs are contraindicated in *SCN1A-* or *SCN2A*-related epilepsy caused by LOF variations [142]. Reversely, the mechanism of pathogenesis is opposite in GOF variations in these genes, and SCBs are recommended [143–145].

**Table 3:** Aetiology-based treatment approaches in monogenic epilepsies

Intervention	Mutated Genes	OMIM Phenotype(s)	Rational	References
Aetiology-based prescription of ASM				
SCBs	*SCN1A*/*SCN2A*/*SCN8A* (GOF variants)	GEFS+ type 2/DEE 6B (non-Dravet)	Inhibit sodium channels with GOF mutations	[143–145]
SCBs	*PRRT2*	Benign familial infantile Seizures 2/Familial infantile Convulsions with paroxysmal choreoathetosis	Mutated PRRT2 may lose its modulatory effect on sodium channels	[157]
Avoid SCBs	*SCN1A* (LOF variants)	Dravet syndrome	The drug target is damaged with LOF changes	[142]
Topiramate	*CACNA1E*	DEE 69	Topiramate also targets CANA1E channels	[158–160]
Ganaxolone	*CDKL5*	DEE 2	Unknown mechanism	[161]
Vigabatrin	*TSC1/TSC2*	TSC/FCD type II	Possible explanation is that epilepsy in TSC arise due to impairment in GABAergic function	[162]
Cannabidiol	*TSC1/TSC2*	TSC/FCD type II	Possible: The drug is a positive allosteric modulator at GABA-A-R which may reverse GABAergic impairment in TSC	[163]
Azetukalner (In Phase 3 CT)	*KCNQ2*	DEE 7/benign neonatal seizures 1	Azetukalner activate potassium channels	[164]
Avoid Valproate	*GLDC*	Glycine encephalopathy1	Valproate inhibits glycine metabolism and worsening pathogenesis	[165]
Avoid Valproate	*OTC*	OTC deficiency	Valproates inhibit urea-cycle enzymes exacerbating underlying hyperammonaemia	[166]
Avoid Levetiracetam	*SVA2*	DEE 113	Mutated drug target cause ineffectiveness and worsening of symptoms	[167]
Targeted aetiology-based therapies				
Rapamycin/ Evirolimus	*TSC1/TSC2*	TSC/FCD type II	Drugs inhibit mTORC1 and reverse hyperactivation of mTORC1 in TSC	[168]
Pyridoxine with lysine restricted diet	*ALDH7A1*	Early-onset Vitamin-B6-dependent epilepsy 4	Deficiency causes toxic lysine metabolites depletes pyridoxine; lysine restriction reduces toxic products of lysine degradation (before 6 months)	[169, 170]
Pyridoxal phosphate (PLP)	*PNPO*	Pyridoxamine 5’-phosphate oxidase deficiency	PNPO synthesizes PLP from pyridoxine so deficiency in the PNPO results in unresponsive to Pyridoxine	[171]
Pyridoxine or PLP	*PLPBP*	Early-onset Vitamin-B6-dependent epilepsy 1	Deficiency of PLP binding protein result in dysregulation of PLP levels	[172]
Uridine	*CAD*	DEE 50	Uridine converted to UMP by uridine kinase restoring deficiency in pyrimidines	[173]
Copper-histidine	*ATP7A*	Menkes disease	Early parenteral supplementation to overcome impaired copper absorption	[174]
Folinic acid (B9)	*FOLR1*	Neurodegeneration due to cerebral folate transport deficiency	Active form of folate	[71, 72]
Serine	*PSAT1*	Phosphoserine aminotransferase deficiency	Early supplementation at birth	[71]
Biotin	*BTD*	Biotinidase deficiency	Biotin is recycled through biotindase hence biotin should be supplemented	[71]
Ketogenic Diet	*SLC2A1*	GLUT1 deficiency syndrome	High fat diet increases ketone bodies which is used instead of glucose as a energy source	[71]
creatine monohydrate	*GAMT/SLC6A8/GATM*	Cerebral creatine deficiency syndrome 1/2/3	supplementation of deficient creatine	[175]
D-Galactose	*SLC35A2*	Congenital disorder of glycosylation type IIm	increase UDP-galastose transport to Golgi and enhance glycosphingolipids levels	[176]
D-ribose	*ADSL*	Adenylosuccinase deficiency	increases de novo purine synthesis/conversion to glucose via the pentose phosphate pathway.	[177]
Protein-restricted diet	*OTC*	OTC deficiency	error of metabolism of the urea cycle	[166]
Enzyme replacement therapy				
Cerliponase alfa	*TPP1*	Neuronal Ceroid lipofuscinosis 2	Replacement of the deficient enzyme	[178]
Aetiology-based decision for epileptic surgery				
Surgery	*DEPDC5*,* NPRL2*,* NPRL3*,* mTOR pathway*	mTORopathies and FCD	These disorders are associated with focal lesion that can be resected in surgery with good postsurgical outcomes	[153]
AVV-vector-mediated gene therapy				
ETX-101 (phase I/II CTs)	*SCN1A*	Dravet syndrome	delivers a transcription factor that explicitly elevates the expression of sodium ion channels (Nav1.1) in GABAergic interneurons	[185]
Antisense oligonucleotides (ASO)				
STK-001 (phase I/II CTs)	*SCN1A*	Dravet syndrome	augments expression of wild-type Nav1.1 subunits in GABAergic interneurons to tackle haploinsufficiency in DS	[185]
Elsunersen (Phase III CTs)	*SCN2A (GOF variants)*	DEE 11	reduces expression of the Nav1.2 protein	[186]

### Aetiology-based targeted treatment

Epilepsy continues to be resistant to polytherapy in some cases despite proper treatment with ASMs. Re-purposed medications can be utilized to tackle the underlying mechanism of epileptogenesis (Table 3). To exemplify, mTOR inhibitors are specifically effective in mTORopathies, including *TSC1* and *TSC2*-related epilepsy [146]. Furthermore, epileptogenesis mechanisms can result in a deficiency of vitamins or other natural substrates in inborn error of metabolism, leading to resistance to ASMs without early correction of the underlying deficiency.

### Surgical interventions in resistant epilepsies

In refractory focal-onset epilepsy (with or without secondary generalization), localized and lateralized epileptic foci can be resected to achieve seizure freedom and, more preferably, ASM discontinuation [147, 148]. Early surgery offers two-dimensional benefits, including seizure control and neurocognitive rescue [148, 149]. Despite the utility of surgery in controlling seizures in intractable cases, it is not suitable or successful in all patients. Therefore, presurgical evaluation results in excluding 75% of evaluated cases [150, 151]. The presence of a confined histopathological lesion in MRI (e.g., HS and FCD type IIb) predicts favourable seizure control and neurodevelopmental improvement [152]. Furthermore, molecular findings can predict the utility of surgery [152–154]. For example, channelopathies and synaptic dysfunction are associated with poor surgical outcomes, while mTORopathies predict good prognosis [153].

### Personalised molecular therapy

The ultimate goal of personalized medicine is to supplement or correct the underlying genetic defect. However, these approaches are in the preclinical development stages or in early clinical trials for genetic disorders in general [155, 156]. One strategy in molecular therapy is delivering deficient genes in LOF variants, causing epilepsy. The adeno-associated virus vector (AVV) is utilized to provide genetic material to the brain through intravenous, intrathecal, or intraventricular routes, or via direct injections into relevant brain tissues. Additionally, the AVV vector can be utilized to deliver transcription factors or modulators that alter the expression of target genes. Another approach in molecular therapy involves targeting mRNA molecules by antisense oligonucleotides resulting in the upregulation or downregulation of target gene expression. Examples of molecular therapies of epilepsy that are in clinical trials are highlighted in Table 3.

## Conclusions

Heterogeneity of Monogenic epilepsies is rooted in their aetiologies, namely pathogenic variations in epilepsy genes. Epilepsy genes participate in divergent pathways regulating complex neuronal function, development and architecture. Besides the putative causal gene, the genetic background plays add further diffraction to the phenotypic spectrum of monogenic epilepsies by altering the severity and drug response. Personalized treatment approaches that address the underlying genetic variations are warranted particularly in cases refractory to the standard medications.

## Future directions

Continuous review and updates in the list of monogenic aetiologies of epilepsy is warranted giving the rapidly expanding literature. In the diagnostic settings, locus heterogeneity in epilepsy phenotypes should be carefully addressed in suspected cases to avoid extensive diagnostic delays, particularly in actionable genetic disorders with available management approaches.

## Supplementary Information

Below is the link to the electronic supplementary material.

Supplementary Material 1 (XLSX 17.0 KB)

Supplementary Material 1 (XLSX 221 KB)

Supplementary Material 1 (XLSX 107 KB)

Supplementary Material 1 (XLSX 191 KB)

## Abbreviations

ASM: Antiseizure medication
DEE: Developmental and Epileptic encephalopathy
GOF: Gain of function
LOF: Loss of function
NDD: Neurodevelopmental disorder
PRS: Polygenic Risk Score
SCB: Sodium channel blocker
